# Structural equation modelling analysis determining causal role among *methyltransferases*, *methylation*, and *apoptosis* during human pregnancy and abortion

**DOI:** 10.1038/s41598-020-68270-1

**Published:** 2020-07-24

**Authors:** Nishat Fatima, Syed Habeeb Ahmed, S. S. Chauhan, Owais Mohammad, Syed Mohd. Fazlur Rehman

**Affiliations:** 1 Interdisciplinary Biotechnology Unit, Aligarh Muslim University (AMU), Aligarh, Uttar Pradesh 202002 India; 2 Department of Biochemistry, All India Institute of Medical Sciences (AIIMS), Ansari Nagar, New Delhi, 110029 India; 3 Department of Biosciences, Jamia Milia Islamia, New Delhi, 110025 India; 4 Department of Surgery, Dr. Ram Manohar Lohia Hospital and Post Graduate Institute of Medical Research Education and Research (PGIMER), New Delhi, 110001 India

**Keywords:** Methylation analysis, DNA methylation, Prognostic markers

## Abstract

The human implantation failure during first trimester leads to spontaneous abortions. Spontaneous abortions are consecutive and occur twice or thrice (with or without prior live births) due to factors which are either maternal or fetal. However, it also constitutes of unknown etiology; known as unexplained recurrent spontaneous abortions (URSA). In this study, the medical terminated human normal early pregnancies (NEP) of the first trimester were taken as control samples, the normal decidual sample whose molecular and epigenetic changes were compared with that of decidua of human URSA subjects. Apoptosis-related genes reported in consecutive recurrent pregnancy loss became the basis for this study. So, in this study, we evaluated the hypothesis that “p53 methylation level through methyltransferases (G9aMT and DNMT1) implicates the fate of embryo towards sustenance or cessation of pregnancy”. Further, the interaction between P53, BAX, BCL-2, CASPASE-6, G9aMT, DNMT-1, and methylated p53 expression level(s) during the first trimester of both URSA and NEP are included in this study. The degree of p53 methylation during the first trimester is found to be significant and positively correlated with that of G9aMT (*p* < 0.05), BCL-2 (*p* < 0.001), and DNMT1 (*p* < 0.001) at both transcript and protein level. A significant and negative correlation (with *p*-value < 0.001) between the degree of p53 methylation during the first trimester and that of the expression level of TUNEL assay (Apoptosis), P53, BAX, and CASPASE-6 are also observed in the present study. A positive correlation between apoptosis and a higher level of p53 expression (which is possibly due to low degree of p53 methylation) is observed both at the transcript and protein level in URSA which is in line with our findings. The analysis performed using structural equation modelling (SEM) further throws light on the causal relationship between sustenance of pregnancy or URSA during the first trimester of a human pregnancy and degree of methylation of p53 which is closely correlated with the interaction between G9aMT, DNMT1, BCL-2, BAX, P53, CASPASE-6, and apoptosis.

## Introduction

Apoptosis is being reported to play a key role during menstruation, parturition, and also during normal pregnancy in both maternal and fetal tissues^1–3^. Trophoblast cell’s immune tolerance against paternal antigens is also suggested to develop due to apoptosis^4–6^. The role of apoptosis and its regulatory pathway is ambiguous in both unexplained recurrent spontaneous abortions (URSA) and pregnancy.

The mystery of embryonic failure due to URSA remains unresolved overall in 24–60% cases^7^. The prevalence of URSA is reported to be 50–75% by ASRM in couples attempting to reproduce with no identifiable causative factors for their pregnancy failures^8^.

URSA is free from any underlying physical, physiological, biochemical, environmental, and genomic factors either in isolation or in combination. This compelled us to enhance our level of understanding and investigate the defects in cellular homeostasis maintained by apoptosis and its epigenetic regulation. Previous studies suggested the involvement of apoptosis to be extremely important for proper functioning of the uterine cells during menstruation, pregnancy and parturition^1–3^. However, the effect of apoptosis and its causation is ambiguous in cases of URSA.

Studies have reported polymorphism of apoptotic gene p53 to not cause any effect in cases of RPL in human^9^. However, the uterine specific p53 deletion in mice has confirmed p53 to be a responsible candidate for 50–60% preterm birth with dystocia and even fetal death in mice^10^. The involvement of other apoptotic molecules Bcl-2, Bax and Caspase-6 are previously being reported in menstruation, RSA, Pregnancy, and Parturition^1,3,11,12^. Whereas not much is reported about bax, bcl-2, caspase-6 and p53 during first trimester of normal early pregnancy (NEP) and URSA.

Due to the absence of p53 genetic mutation and presence of its gene expression in URSA, epigenetic causes like methylation of the p53 gene was taken into account for this study. However, the expression of apoptotic gene(s)/protein(s) like bax, bcl-2, caspase-6 and p53 is also evaluated in this study.

The mechanism of methylation is known to precede the regulatory events both at genome and proteome level. Methyltransferases are the molecules involved in methylation of genes and plays a crucial role in genetic regulation. DNA methylation and histone modification are the two most cardinal epigenetic mechanism that arbitrate gene expression. The methyltransferases (MT) like G9aMT and DNMT1 plays a critical role in methylation. Our earlier study on G9a and H3K9 methylation have rightly indicated its role in URSA^13^. Literature suggests G9aMT alone cannot maintain the normal DNA methylation/silencing and functions only in conjunction with DNMT1 and any loss in it could alter methylation despite of G9aMT presence^14^. Aberration of Gene G9a leads to embryonic lethality during early embryogenesis, hence presence of both DNMT1 and G9aMT are essential for normal embryogenesis and methylation^15^. This information compelled us to include both G9aMT and DNMT1 in our study.

Correlation between parameters can infer to their occurrence together but not causation. Hence, G9a and DNMT1 may be correlated but cannot indicate its involvement in causation of p53 methylation and apoptosis during NEP and URSA. To determine causation, a general methodology is used known as Structural Equation Modelling (SEM) to address complex systems involving many biological networks, genome-wide association studies (GWAS), gene-environment interactions, and linage analysis i.e., quantitative trait loci (QTL)^16–22^. In this study, the biological pathway is selected for SEM as per the a priori genetic knowledge to build a model. The shortest path is considered and the model is fitted with SEM along with its improvement by balancing data and prior knowledge evidences. After multiple pathway analysis, the most significant pathway is considered through SEM which might be taken as the best fit for determining causal relationship among methyltransferases, apoptosis, and methylation during pregnancy and URSA.

Thus, in this study we will be testing and proving our hypothesis i.e., “p53 methylation level through methyltransferases implicates in the causal fate of embryo towards sustenance or cessation of pregnancy”.

## Materials and methods

### Study samples

Fresh Human decidual endometrial tissue (DET) of first trimester were collected of the last curettage soon after the dilatation and curettage (D&C) procedure was performed by Gynecologist in the operation theatre under strict sterile/aseptic conditions for the cases of Unexplained recurrent spontaneous abortion (URSA) and medical termination of unwanted normal pregnancy (NEP) done for population control. As per the American College of Obstetrics and Gynaecology (ACOG)^23^ URSA cases considered in our study comprised of two or more ***consecutive abortions*** (with or without any previous live births) prior to 12 weeks of gestation (i.e., First trimester). DET- samples of 12 NEP and 15 URSA (3-primary, and 12-secondary- URSA) (Table 1)^13,24^ subjects were taken and divided into four parts. The first three portions of tissue was transferred in three different autoclaved vial and snap frozen in liquid nitrogen after which it was stored at − 80 °C for DNA (MS-PCR), RNA (RT-PCR) and protein analysis (western blot)^13,24^. The fourth part of the DET-sample was thoroughly washed with sterile normal saline solution stored at room temperature in fourth autoclaved vial containing 10% buffered formalin at room temperature, for immunohistochemical (IHC) analysis^13,24^. All these samples were collected as per international standards^25^ and specific inclusion–exclusion criteria as provided in Supplementary information of our earlier work. We have excluded Subjects^13,24^.

- Aged less than 20 years or more than 30 years.
- Positive for HIV antibody.
- Suffering or with the symptom of urogenital infection.
- Early uterine pregnancy due to failure of hormonal/steroidal contraceptives.
- Suffering from menstrual/hormonal irregularities.
- Associated with congenital/ traumatic/ anatomical abnormalities, malnutrition or with other diseases like Tuberculosis/diabetics/hypertension/typhoid/pyrexia of unknown origin (PUO).
- Who have attempted any other means of abortion.
- Who have attempted artificial reproductive means for pregnancy.
- Undergoing surgical abortions (D&C procedure) for uterine pregnancy of more than 12th week duration.
- Who had developed any significant disease, such as pre-eclampsia, eclampsia or having a history of drug intake such as prostaglandin, acetylsalicylic acid, and antibiotics.
  The criteria of inclusion for Normal Early pregnancy were^13,24^:
- Subjects with unwanted uterine pregnancy (under MTP act) of less than 12 weeks may or may not be due to failure of contraception devices like condom, intrauterine device, etc.
- Early pregnancy not manifested as threatened/inevitable or incomplete abortion.
  The criteria of inclusion for Unexplained recurrent abortion Early pregnancy were^13,24^:
- Subjects undergoing dilatation & curettage (D&C) due to inevitable/ incomplete abortion of less than 24 h duration for uterine pregnancies of up to 12 weeks without visible evidence of any embryonic abnormality (like hydatidiform moles, etc.,).
- Subjects having history of two or more such types of spontaneous consecutive recurrent abortions*.*

**Table 1:** Details of Collected Samples^13^.

Sample no. of subject	Age of subject	No. of live birth	No. of previous spontaneous abortion (RSA) or MTP (NEP)		Duration of present pregnancy (in weeks)	Surgical abortion by D&C for		
						NEP	URSA-EP	
			RSA (consecutive abortions after live birth (if any))	NEP			Incomplete	Inevitable
N1	26	2	0	0	4	✓		
N2	22	0	0	1	9	✓		
N3	20	0	0	0	12	✓		
N4	29	2	0	0	4	✓		
N5	28	0	0	0	9	✓		
N6	22	3	0	0	11	✓		
N7	28	2	0	0	9	✓		
N8	25	3	0	0	6	✓		
N9	28	3	0	0	10	✓		
N10	30	1	0	0	8	✓		
N11	22	0	0	1	8	✓		
N12	23	1	0	1	4	✓		
R13	27	0	2	0	6			✓
R14	30	0	4	0	12		✓	
R15	30	2	4	0	6			✓
R16	23	1	2	0	10			✓
R17	29	1	2	0	8			✓
R18	28	2	3	1	6		✓	
R19	25	2	2	0	8		✓	
R20	28	1	2	0	8			✓
R21	28	2	2	0	6		✓	
R22	20	1	2	0	12			✓
R23	25	1	3	1	6			✓
R24	22	2	2	0	10			✓
R25	30	3	2	0	6			✓
R26	22	0	2	1	10		✓	
R27	21	2	2	0	12		✓	

N-NEP sample; R-URSA sample.

### IHC studies

IHC is elaborated in our earlier publication^13^ and was performed for all proteins DNMT1, Bcl-2, Bax, Caspase-6, p53 and G9aMT^13^. A semi-quantitative assessment method was used, as described in earlier studies^26,27^ an average of five fields was observed for each specimen, as recommended by De Falco^1^. All values were expressed as Means ± SEM, and differences were compared by using Student’s t-test^28^. The advantage of IHC is the precise localization of the protein on the placental/decidual/endometrial section^24^.

### SDS PAGE analysis

Proteins in the soluble fraction of the tissue extracts were separated under reducing conditions in 12% sodium dodecyl sulphate-polyacrylamide gel electrophoresis (SDS-PAGE) according to a published method^29^. The 15 µl samples containing 30 µg of proteins in threefold-concentrated Laemmli solution (200 mmol/l Tris, pH 6.9, 6% SDS, 6% ß-Mercaptoethanol, 45% glycerol and 0.03% bromophenol blue) were boiled for 10 min before being loaded into the well. After electrophoresis, the proteins were stained with Coomassie brilliant blue, or silver staining was performed or kept unstained in transfer buffer containing 20% methanol for western blot analysis.^24^.

### Western blot

Protein was extracted from the maternal portion decidual endometrial tissue^13^. About 3 mg of wet decidual tissue was processed through the method described by Berkova^30^. Ten micrograms of protein quantified by the Lowry method^31^. Proteins were separated by sodium dodecyl sulfate (SDS)–polyacrylamide gel electrophoresis (PAGE) and transferred to Polyvinylidene difluoride (PVDF) membranes by electro-blotting and reacted with the primary antibodies to rabbit antihuman Bcl-2 polyclonal antibody (B.D. Biosciences) or rabbit antihuman Bax polyclonal antibody (B.D. Biosciences) or rabbit antihuman p53 polyclonal antibody (B.D. Biosciences) or goat antihuman DNMT1 polyclonal antibody (Lifespan Biosciences Inc., LS-B4353) or rabbit anti human G9aMT^13^ (Sigma) at a dilution of 1:50 with 0.5% skimmed milk in Tris-buffered saline (pH 7.5). The membranes were then immersed in the reaction buffer containing peroxidase-conjugated isotype-matched non-immune goat IgG with 0.5% skimmed milk. The reacted bands were developed with a hydrogen peroxide and DAB. Reacted bands of G9aMT^13^ and DNMT1 were scanned, and band intensities were quantified with Chemi-Imager IS-4400 (Alpha Innotech Corp., San Leandro, CA). Statistical analysis was carried out by independent t-test with the SPSS software (version 9.01, SPSS, Chicago)^13,24^.

### Tunnel assay

TUNEL assay was carried out essentially as described previously by Baldi et al. (2000)^32^. After removal of paraffin with xylene and rehydration in ethanol solutions of decreasing concentrations, sections were digested for 10 min with proteinase K (20 mg/ml), washed in distilled water, and exposed briefly to 3% H_2_O_2_ to inactivate endogenous peroxidase. The TUNEL reaction was performed using the peroxidase-based Apoptag kit (Oncor). TUNEL positive cells were detected with diaminobenzidine (DAB) and H_2_O_2_ according to the supplier’s instructions. Finally, stained sections were lightly counterstained with haematoxylin. Cells were defined as apoptotic if they were TUNEL-positive. This experiment was repeated on several different sections for each specimen, obtaining similar results^24^.

The number of apoptotic cells were categorized as (−), < 3 apoptotic cells/field; (+), 3–8 apoptotic cells/field; (+ +), > 8 apoptotic cells/field, at X 630 magnification with use of an arbitrary scoring system comparable with that used by Watanabe et al. (1997)^24,33^.

### RT-PCR

Reverse transcription-polymerase chain reaction (RT-PCR) for DNMT1, Bcl-2, Bax, G9a, p53 and β-actin, was done with cDNA synthesized from the total RNAs of the Normal early pregnancy and URSA decidual endometrial tissue sample. The primer sequences are as follows:

- *dnmt1* (335 bp)-5′-TAT CCG AGG AGG GCT ACC TG-3′; 5′-TGT GAT GGT GGT TTG CCT GG-3′.
- *bcl-2* (390 bp)-5′-GAC TTC GCC GAG ATG TCC AG-3′; 5′-TCA CTT GTG GCT CAG ATA GG-3′.
- *bax* (445 bp)-5′-TTT TGC TTC AGG GTT TCA TCCA-3′; 5′-GAC AGG GAC ATC AGT CGC TT-3′.
- *g9a*^13^ (285 bp)- 5′ –GAG GTG TAC TGC ATA GAT GCC-3′; 5′-CAG ACG GCT CTG CTC CAG GGC-3′.
- *p53* (156 bp)-5′- GGC CCA CTT CAC CGT ACT AA-3′; 5′-GTG GTT TCA AGG CCA GAT GT-3′.
- *β-actin* (442 bp)-5′-CAG CCA TGT ACG TTG CTA TCC AG-3′; 5′-GTT TCG TGG ATG CCA CAG GAC-3′.

### Methylation-specific polymerase chain reaction (MS-PCR)

We have optimized laboratory protocol for bisulphite conversion of the isolated DNA which was then subjected to PCR for checking the expression of both methylated and unmethylated p53. Isolation of genomic DNA and treatment with sodium bisulfite were done according to protocols optimized in our laboratory. After overnight incubation DNA was purified using Wizard DNA purification resin according to the manufacturer’s instructions (Promega)^24^.

Bisulphite treated genome DNA was analyzed by MSP using a primer specific for unmethylated p53 (P1 = 5′-TTG GTA GGT GGA TTA TTT GTT T-3′; P2 = 5′-CCA ATC CAA AAA AAC ATA TCA C-3′, 247 bp) and methylated p53 (P1 = 5′-TTC GGT AGG CGG ATT ATT TG-3′; P2 = 5′-AAA TAT CCC CGA AAC CCA AC-3′, 193 bp) were given the PCR conditions as given below (Table 2)^34^.

**Table 2:** PCR condition for methylation specific PCR.

Gene	Initial denaturation	Denaturation	Annealing	Extension	Cycles	Final extension	Termination
P53 (U)	95 °C; 5 min 80 °C ; 3 min	95 °C , 30 s	58 °C ; 30 s	72 °C ; 30 sec	35	72 °C ; 5 min	4 °C ; 12 h
P53 (M)	95 °C ; 5 min 80 °C ; 3 min	95 °C , 30 s	60 °C ; 30 s	72 °C ; 30 s	35	72 °C ; 5 min	4 °C ; 12 h

The PCR samples were resolved by electrophoresis in a 2% agarose gel and stained with ethidium bromide. Negative control was taken in which the template i.e., bisulphite treated DNA was not added. The genomic DNA from human DETS with normal pregnancy was treated as positive control.

### Statistical analysis

#### ROC curve analysis^13^

The sensitivity (Sn) and specificity (Sp) for the genes were quantified as per the receiver operating characteristic (ROC) curves. The relationship between the protein expression and clinico-pathological parameters were tested by χ^2^ and Fischer’s exact test. Two-sided *p*-values were calculated and *p* ≤ 0.05 was considered to be significant^24,35^.

#### Structural equation modelling (SEM)

We examined the association between latent-methyltransferases (G9aMT & DNMT1), *p53* methylation, and apoptosis using SEM in SPSS-(AMOS) for descriptive statistics, confirmatory factor analysis-structural equation modelling (CFA-SEM), and path analysis.

### Ethics statement

All the mandatory ethical permissions were obtained from SafdarJung Hospital (New Delhi, India) and informed written consent from each individual subjects were taken for conducting this study. Also, all methods were performed in accordance with the relevant guidelines and regulations and were approved by the Interdisciplinary Biotechnology Unit, A.M.U, Aligarh, India^24^. The NEP tissue samples and the URSA tissue samples were matched according to relevant clinical parameters.

## Results

### Clinical history of subjects

The patient’s history and parameters are shown in (Table 1), the inevitable and incomplete abortions were categorized under URSA with both primary and secondary aborters having more than 1 previous abortion history and aged ≤ 30 years. The subjects suffered inevitable/incomplete abortions were mostly secondary aborters with prior history of live births as well as multiple unknown abortions during the first trimester.

### Transcript expression of methylation and apoptosis related genes

**Figure 1:**
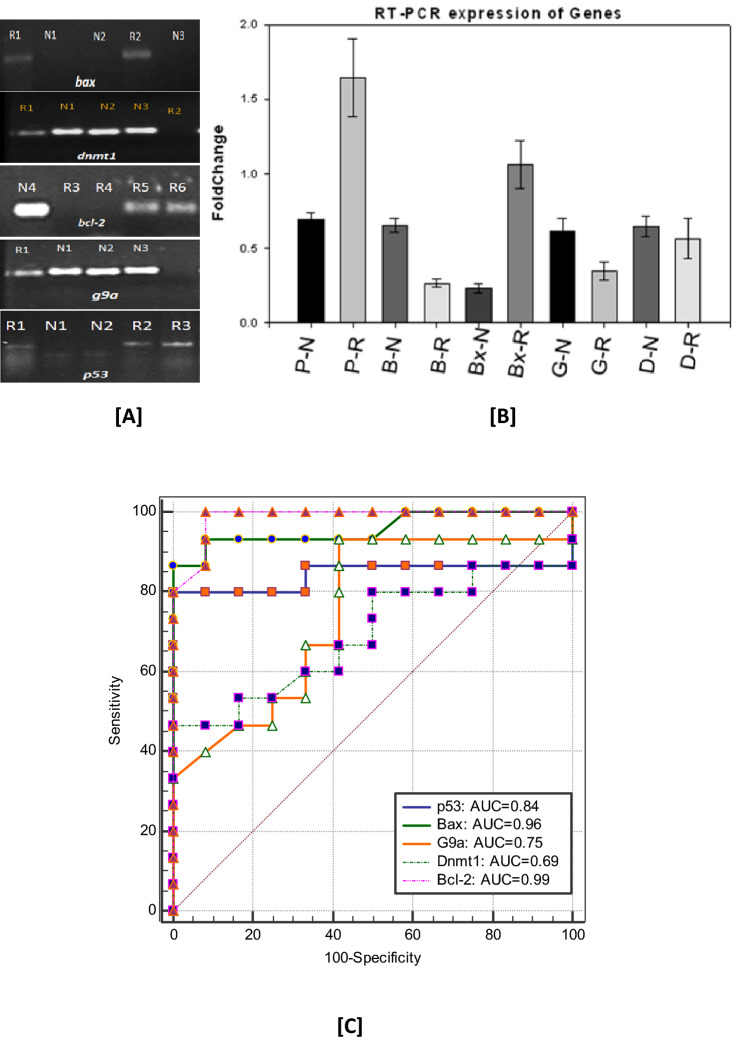
(**A**) RT PCR analysis of the DET showing cDNA expression of Bcl-2 (B), Bax (Bx), G9aMT (G), DNMT1 (D) and p53 (P) in URSA (R) and NEP (N). (**B**) Error bar chart showing the fold change values of different genes in the DET of R & N. (**C)** ROC curves of respective upregulated/downregulated transcript during the first trimester of NEP and URSA.

The most crucial step of central dogma comprises of two step i.e., transcription and translation. Transcription being an initial stage for transfer of information from DNA to RNA is evaluated in this study. The embryo comprises of indispensable mRNAs during the early stages of development and proper molecular milieu is essential for it to proceed normally. The NEP cases compared to URSA showed upregulated methylation related transcripts (dnmt1 and g9a) and anti-apoptotic transcripts (bcl-2). Whereas, URSA cases showed upregulated apoptotic transcripts (bax and p53) (Fig. 1A,B). The fold change and Standard Error of Mean (SEM) is given in Table 3 of all transcripts.

**Table 3:** Evaluation of genes in DETS for a panel of genes.

Gene(s)	RT-PCR (Fold change ± SEM)			RT-PCR-ROC-URSA-DETS					
	NEP	URSA	*p*-value	Sensitivity (%)	Specificity (%)	AUC	SE	95%CI	*p*-value
G9a	0.61 ± 0.09	0.34 ± 0.06	0.016	66.67	66.67	0.75	0.09	0.55 to 0.89	0.028
Dnmt1	0.65 ± 0.07	0.57 ± 0.13	0.612	58.33	60	0.69	0.11	0.49 to 0.86	0.08
Bcl-2	0.66 ± 0.05	0.27 ± 0.03	< 0.001	91.67	93.3	0.99	0.02	0.85 to 1.00	< 0.0001
Bax	0.23 ± 0.03	1.06 ± 0.16	< 0.001	91.67	93.3	0.96	0.04	0.79 to 0.99	< 0.0001
P53	0.69 ± 0.04	1.64 ± 0.26	0.003	83	80	0.84	0.08	0.65 to 0.95	0.002

The sensitivity and specificity of all the transcripts mentioned in Table 3 and Fig. 1C along with AUC of ROC analysis is significant in the Decidual Endometrial tissue system (DETS)and may be used as a potential biomarker for diagnosis/prognosis of URSA.

### Protein expression of methylation and apoptosis related proteins

**Figure 2:**
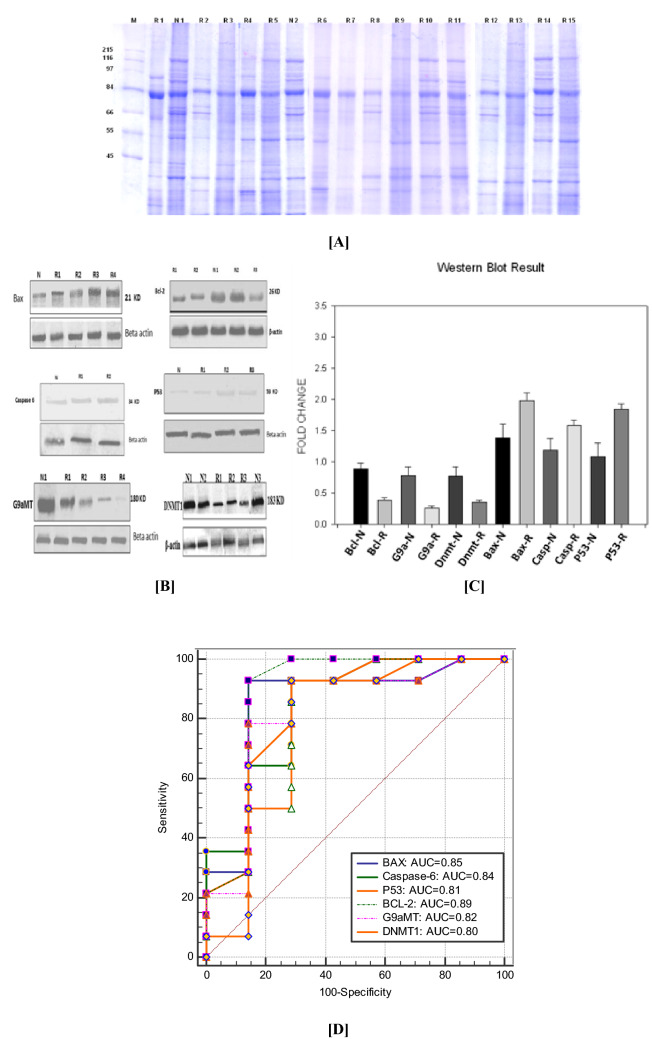
(**A**) SDS PAGE of the protein extracted from DETS of URSA and NEP showing differential expressions. (**B**) Western blot analysis of DET showing different protein expression of NEP (N) and URSA(R) cases of BAX, BCL-2, P53, CASPASE-6, G9aMT, and DNMT1. (**C**) Error bar chart showing the fold change values of different protein expression of methylation and apoptosis related proteins in both URSA (R) and NEP (N). (**D**) ROC curves demonstrating the respective upregulated proteins in both URSA and NEP.

**Figure 3:**
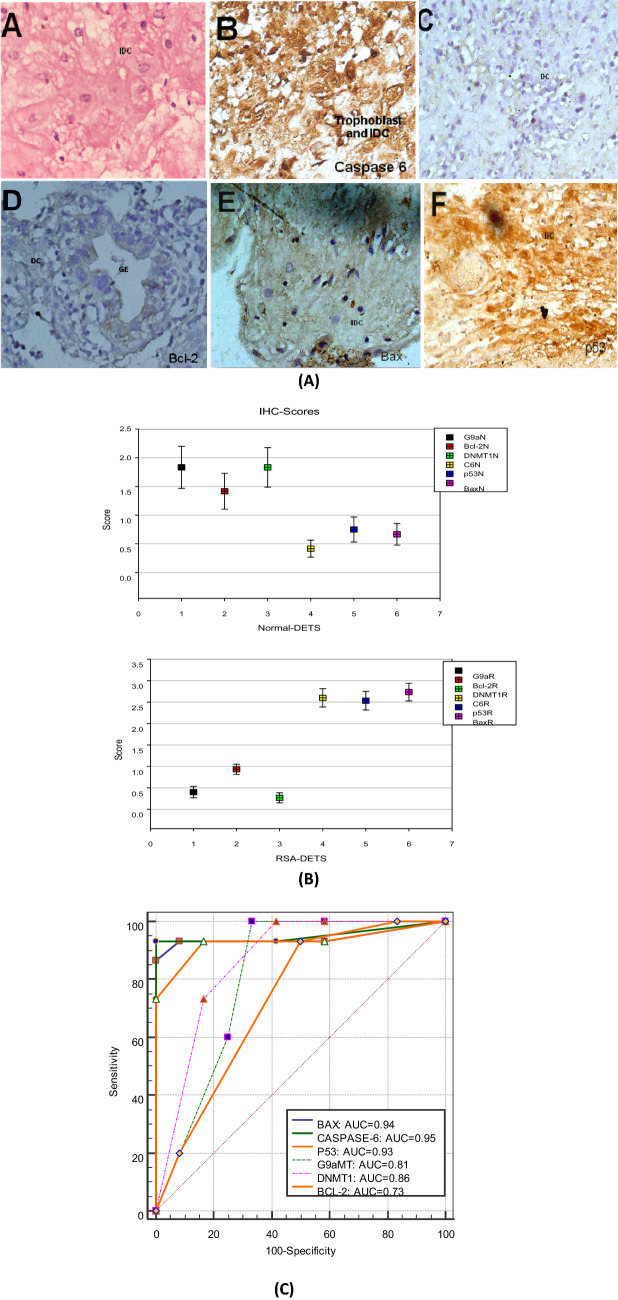
(**A**) Immunohistochemical analysis of various apoptosis related protein in NEP and URSA decidual/endometrial tissue system (A). Histopathological analysis performed through H&E staining shows Inflammed decidual cells (IDC) and trophoblast cells in URSA decidual/endometrial tissue sections. (B). Caspase 6 Positive Immunoreactivity observed in the trophoblast cells as well as cytoplasm and nuclei of decidual cells in URSA tissue sections. (C) No Caspase 6 immunoreactivity in the tissue section of NEP cases. (D) Bcl-2 low immunoreactive decidual cytoplasm and glandular (GE) observed in NEP tissue sections; whereas no reactivity observed in URSA. (E) Bax positive immunoreactivity in IDC and cytoplasm was observed but no immunoreactivity in nucleus of URSA DC was demonstrated nor in NEP decidual/endometrial tissue sections. (F) p53 positive immunoreactivity in DC of URSA. (**B**) Graph representing the IHC score of NEP and URSA DET showing lower expression of G9aMT, Bcl-2 and DNMT1 and higher expression of Caspase 6, p53 and Bax in the URSA-DET. (**C**) ROC curve graph of IHC score of all the molecules in URSA and NEP.

The NEP and URSA protein expression observed by both western blot (Fig. 2, Table 4) and immunohistochemistry (Fig. 3, Table 5) were in congruence to that of the results of transcript expressed. The methylation (DNMT-1 & G9aMT) and anti-apoptotic (BCL-2) proteins were upregulated in NEP compared to that of URSA. Whereas, apoptotic (BAX, CASPASE-6 and P53) were upregulated in URSA compared to that of NEP.

**Table 4:** Evaluation of proteins in DETS through western blot.

Protein(s)	WB-[Fold change ± SEM]			URSA-DET					
	NEP	URSA	*p*-value	Sensitivity (%)	Specificity (%)	AUC	SE	95% CI	*p*-value
G9aMT	0.94 ± 0.15	0.33 ± 0.07	0.002	78.57	85.71	0.82	0.12	0.59 to 0.95	0.006
DNMT1	0.81 ± 0.11	0.41 ± 0.05	0.004	92.86	71.43	0.80	0.13	0.57 to 0.94	0.024
BCL-2	1.34 ± 0.17	0.43 ± 0.06	< 0.001	92.86	85.71	0.89	0.11	0.67 to 0.98	0.0003
BAX	1.22 ± 0.12	1.91 ± 0.13	0.007	92.86	85.71	0.85	0.11	0.63 to 0.97	0.001
P53	1.21 ± 0.13	1.78 ± 0.10	0.007	92.86	71.43	0.81	0.12	0.58 to 0.94	0.012
CASPASE-6	1.06 ± 0.10	1.55 ± 0.08	0.004	92.86	71.43	0.84	0.10	0.62 to 0.96	< 0.001

**Table 5:** Evaluation of proteins in DETS through IHC.

Protein(s)	IHC (Fold change ± SEM)			IHC-ROC-URSA-DET					
	NEP	URSA	*p*-value	Sensitivity (%)	Specificity (%)	AUC	SE	95% CI	*p*-value
G9aMT	1.83 ± 0.37	0.40 ± 0.13	< 0.001	75	60	0.81	0.09	0.612–0.933	0.007
DNMT1	1.83 ± 0.34	0.27 ± 0.12	< 0.001	83.33	73.33	0.86	0.08	0.674–0.963	0.002
BCL-2	1.58 ± 0.26	0.87 ± 0.13	0.015	50	93.33	0.73	0.10	0.529–0.884	0.040
BAX	0.67 ± 0.19	2.73 ± 0.21	< 0.001	91.67	93.33	0.94	0.05	0.783–0.996	< 0.0001
P53	0.75 ± 0.22	2.60 ± 0.22	< 0.001	83.33	93.33	0.93	0.05	0.763–0.992	0.0001
CASPASE-6	0.42 ± 0.15	2.60 ± 0.21	< 0.001	93.33	100	0.95	0.05	0.795 to 0.998	< 0.0001

### Methylation affecting the apoptotic pathway

In order to clarify the epigenetic regulation, we have studied both gene and histone methylation. Although previously we published histone methylation results^13^, in this study we evaluated P53 gene methylation through methylation specific PCR in both NEP and URSA cases.

**Figure 4:**
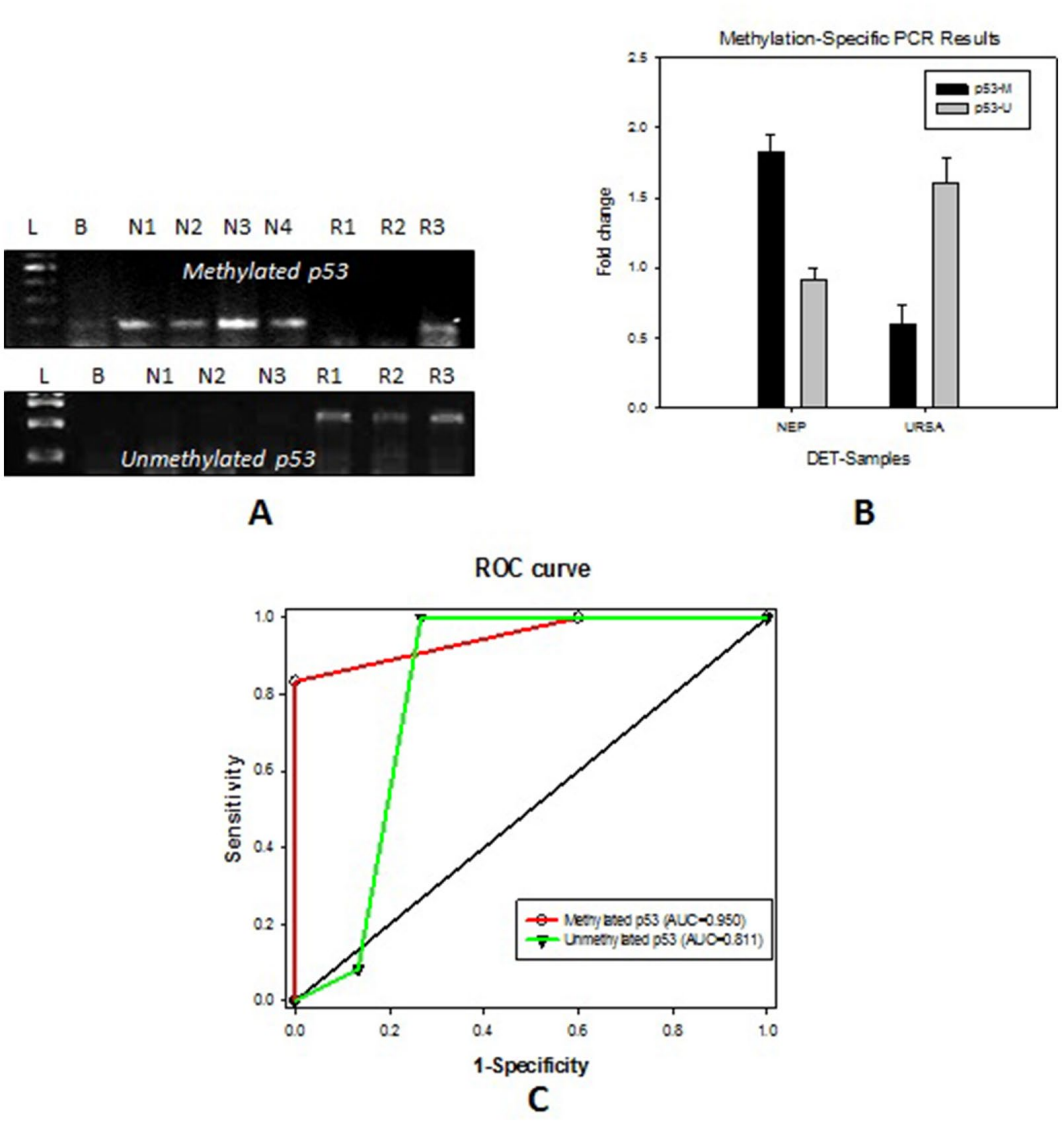
(**A**) Expression analysis of methylated (193 bp) and unmethylated (247 bp) p53  in the DET of URSA and NEP through MSP (L= Ladder; B = Negative control; R = URSA; N = NEP). (**B**) Fold change observed in URSA and NEP- DET of methylated and unmethylated p53. (**C**) ROC curve analysis of Methylated and unmethylated p53 representing the respective Area Under the curve (AUC).

Upregulated methylated p53 is observed in NEP compared to URSA, whereas, increase in unmethylated p53 is observed in URSA compared to NEP (Fig. 4).

**Figure 5:**
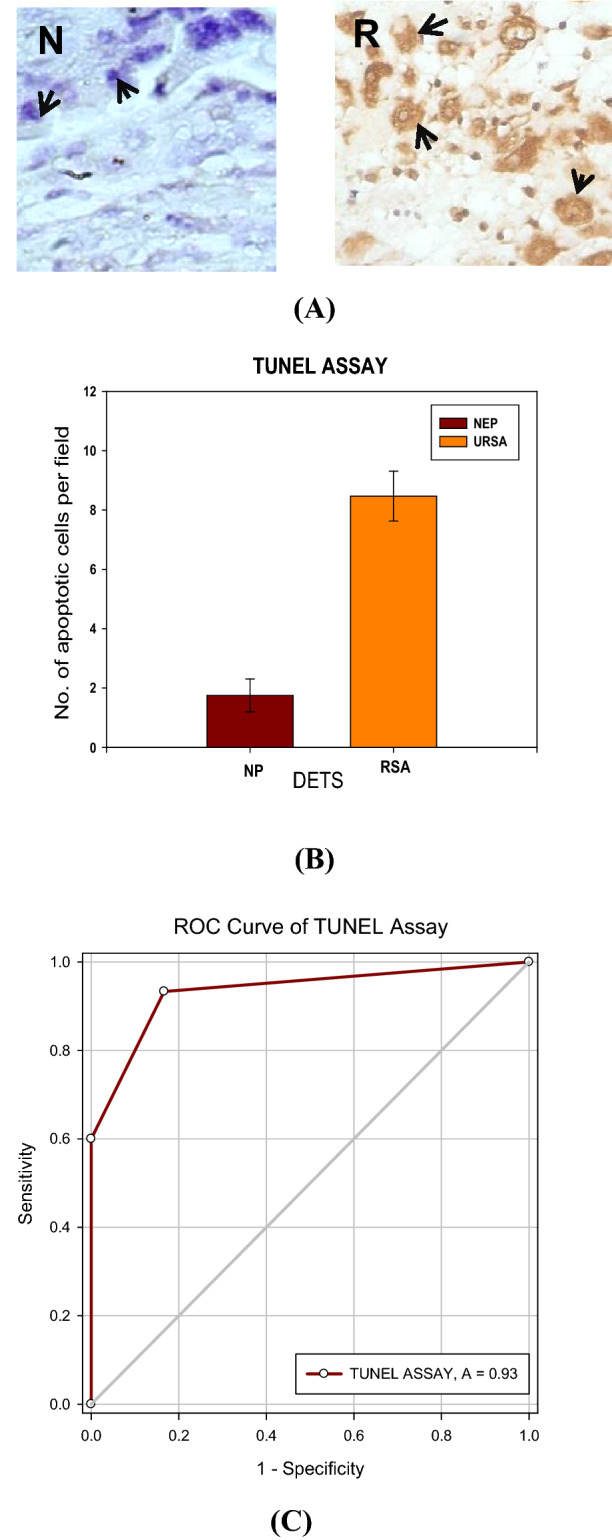
(**A**) Representative Photomicrograph of TUNEL assay of histological sections of DET from NEP and URSA cases showing unstained nuclei (NEP) and stained nuclei (URSA) showed apoptosis.[Arrows showing nucleus]; (**B**) The graph representing more no. of apoptotic cells in the URSA as compared to the NEP. (**C**) ROC curve graph of TUNEL assay.

As p53 gene is associated with apoptotic pathway, the samples were subjected to TUNEL assay. Lower no. of apoptotic cells observed in NEP compared to that of URSA-DET along a very statistically significant *p*-value of 0.000001 (Fig. 5).

The results suggest high p53 methylation level and low apoptosis level in NEP in contrast to low p53 methylation level and high apoptosis level in URSA.

### Suggestive diagnostic/prognostic marker

The ROC plot, represents fundamental ability of a test to discriminate between two states of health (i.e., diseased and normal) and is an index of pure accuracy. A nonparametric ROC plot is an unbiased view of a test’s performance (accuracy) in a defined clinical setting. Hence this ROC plot analysis is performed for the most significant molecular marker which could be a used either as diagnostic/prognostic purpose. Also other aspect is to evaluate the most reliable technique to detect the marker i.e., PCR, Western blot, or IHC.

Hence, Sensitivity and specificity are the measure of accuracy in correctly diagnosing the presence and absence of condition respectively. Whereas, Area under the ROC curve (AUC) is a measure of how well a parameter can distinguish between two diagnostic groups (diseased/normal).

Higher AUC, sensitivity, and specificity is observed in IHC for all the proteins (Fig. 3C; Table 5). Whereas, *p53, bax, bcl-2* genes showed higher values obtained through RT-PCR (Fig. 1C; Table 3). The MS-PCR and TUNEL both showed a higher AUC (Figs. 4 and 5).

### Causal relation between methylation and apoptosis

To address the relationship between methyltransferases, methylation and apoptosis during early normal pregnancy and abortion, we used structural equation models (SEMs) of SPSS-AMOS. The SEM model was created, taking into account the hypothesis.

**Figure 6:**
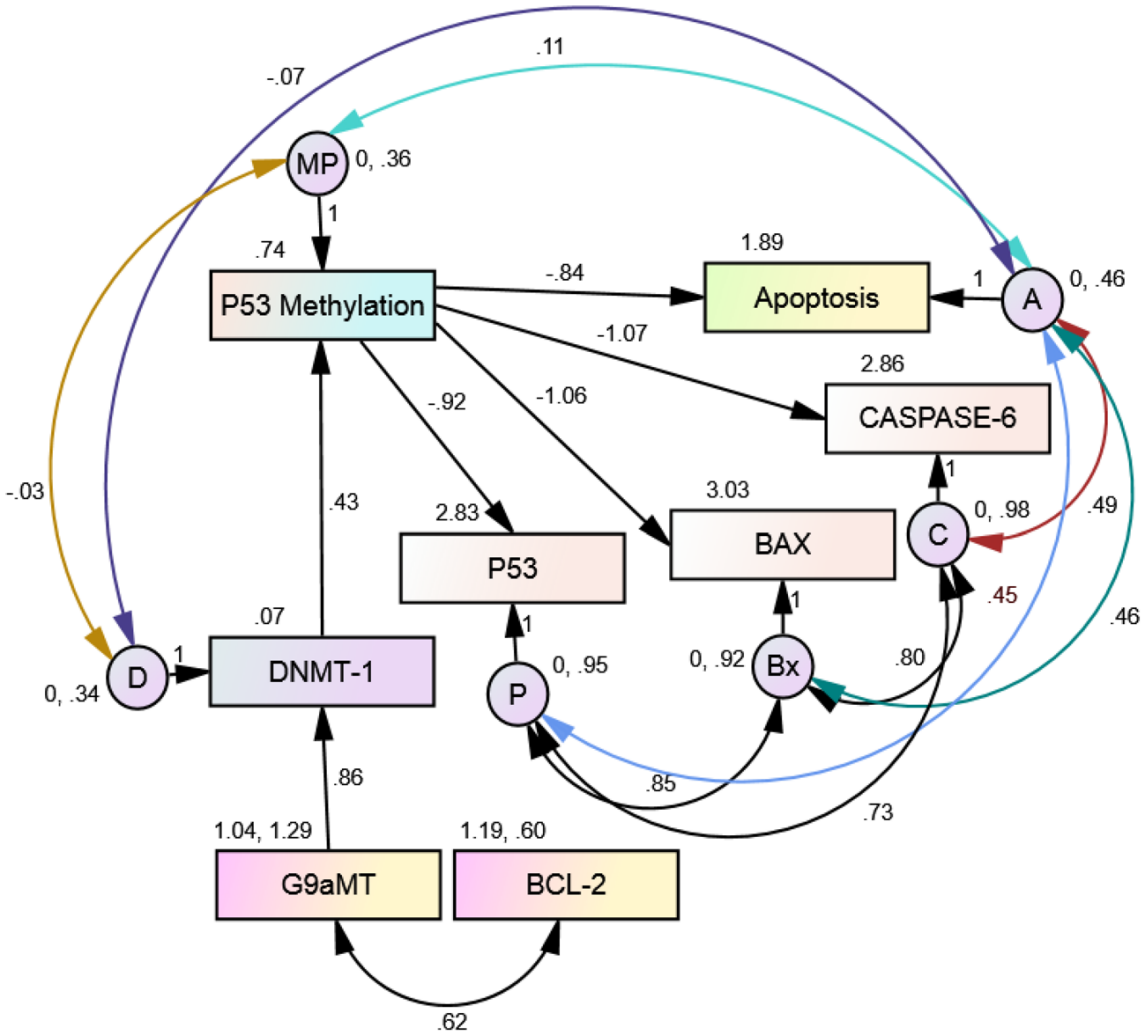
Causal relationship pathway.

The created model shows consistency with the observed results through different tests. The observed variables in relationship with the latent construct comprised of the minimum discrepancy divided by the degrees of freedom (CMIN/DF) as 0.929, 0.04 root mean square residual (RMR), < 0.0001 Root mean square error of approximation (RMSEA), 0.95 Normed fit index (NFI), 0.89 (RFI), 1.00 Incremental fit index (IFL), 1.00 Comparative fit index (CFI), and Tucker-Lewis index (TLI) is 1.01. Factors were dropped until the fit and validity were acceptable, these results depict the model to be a good fit, and all of the variables significantly loaded onto the latent factors; Also, the factors were highly correlated in the model significantly (Fig. 6, Tables 6, 7).

**Table 6:** Estimates from final CFA model.

	B	S.E	C.R	*p*	b
DNMT1 <-- G9aMT	0.859	0.1	8.559	***	0.859
p53 Methylation <-- DNMT1	0.428	0.12	3.558	***	0.645
p53 <-- p53 Methylation	− 0.916	0.253	− 3.613	***	− 0.578
TUNEL <-- p53 Methylation	− 0.839	0.25	− 3.363	***	− 0.743
BAX <-- p53 Methylation	− 1.055	0.249	− 4.231	***	− 0.639
Caspase 6 <-- p53 Methylation	− 1.072	0.258	− 4.162	***	− 0.632

B = unstandardized beta, b = standardized beta; ***p < 0.001.

**Table 7:** Total standardized direct and indirect effects of CFA model.

	G9aMT		DNMT1		p53 Methylation	
	Direct	Indirect	Direct	Indirect	Direct	Indirect
DNMT 1	0.859	0	0	0	0	0
p53 Methylation	0	0.554	0.645	0	0	0
TUNEL	0	− 0.411	0	− 0.479	− 0.743	0
Caspase 6	0	− 0.35	0	− 0.408	− 0.632	0
P53	0	− 0.32	0	− 0.373	− 0.578	0
BAX	0	− 0.354	0	− 0.412	− 0.639	0

The exogenous factor G9aMT is found to exhibit a positive relationship with DNMT1, later being an endogenous factor is effective only once stimulated by G9a. Whereas, G9aMT is being influenced by BCL-2 which is yet another exogenous factor positively correlated with it.

The combined effect of G9aMT, DNMT1 and BCL-2 leads to methylation of p53 which negatively influences the other endogenous factors P53, BAX, CASPASE-6 and apoptosis (assessed by TUNEL assay).

Hence, the results clearly depict causal relationship between methylation enzymes (G9aMT & DNMT1), p53 methylation and apoptosis during pregnancy and abortion.

## Discussion

The causal relationship is depicted through Structural equation modelling (SEM) in this study. SEM results depicts the effect of BCL-2 on methyltransferases G9aMT that has a direct effect on DNMT1 ensuing p53 methylation during normal pregnancy. The *p53* gene methylation also shows significant direct negative correlation with p53, bax, and caspase-6; Also, TUNEL results are negatively correlated with p53 methylation (Fig. 6, Tables 6, 7). These results are consistent to the findings of other group, which affirms cre-mediated deletion of dnmt-1 ensuing p53-dependent apoptosis^36^.

Earlier studies in the field of reproductive research has proved the role of apoptosis in causing menstruation and normal child birth in the late pregnancy^3^. But the role of apoptosis and p53 methylation was not explored in the cases of early pregnancy especially in the cases of abortions. This missing link was recognized by us and the study have been planned to explore the role of methyltransferases, p53 methylation, and apoptosis in the cases of early pregnancy and in the cases of unexplained abortion during early pregnancy^24^.

A study conducted by Haidacher and group^37^ suggested that p53 is over expressed not only in malignant tumour cells but in certain trophoblast cell populations of the human placenta as well i.e., of term pregnancy. Smith et al.,^38^ showed significant increased placental apoptosis as the pregnancy progresses, suggesting that it may play a role in the normal development and aging of the placenta. Whereas our study suggests that if this increase in apoptosis is observed in an earlier stage of pregnancy during the first trimester it results into URSA^24^.

Methyltransferases are known to be bound with methylation activities, but its regulation and trigger in pregnancy is yet to be ascertained. The role of methyltransferases like G9aMT and DNMT1 is meagerly understood for its correlation with the etio-pathogenesis of URSA. Through our study we found expression of both DNMT1 and G9aMT to be expressed normally during NEP helping in maintaining a pregnancy whereas a down regulation in their expression was observed in the DETS resulting into URSA during first trimester. This study also shows an anti-apoptotic protein BCL-2 to be positively correlated with expression of G9aMT.

Consistent results are observed with western blot, RT-PCR, and IHC for all the genes and proteins. The ROC curve has been plotted to find out the grading of the undertaken tests to be in the order IHC, RT-PCR and WB with respect to various molecular factors to identify URSA. The IHC-PCR-ROC curve shows the effectiveness of the individual molecules in decreasing order- CASPASE-6 (Sp-100%, Sn-93.33%, AUC-0.95), BAX (Sp-93.33%, Sn-91.67%, AUC-0.94), P53 (Sp-93.33%, Sn-83.33%, AUC-0.93), DNMT1 (Sp-73.33%, Sn-83.33%, AUC-0.86), G9aMT (Sp-60%, Sn-75%, AUC-0.81), BCL-2 (Sp-93.33%, Sn-50%, AUC-0.73) (Fig. 3C, Table 5).

The results of PCR, IHC and western blot has confirmed the reciprocal relationship in the expression of anti-apoptotic (BCL-2, G9aMT, and DNMT1) versus apoptotic (p53, BAX, and CASPASE-6) gene(s)/protein(s), which is in accordance with the results of previous researchers in the field^36,39–42^, and no literature could be traced in contravention till date^24^.

The reciprocal relationship through Immunohistochemistry could be explained by the elevated expression of G9aMT, DNMT1 and Bcl-2 while low expression level of p53, Caspase 6 and Bax were observed in NEP whereas the reverse occurs during URSA. Also, TUNEL positive nuclei were observed in the URSA cases of first trimester to be a reciprocal finding as observed in previous study^1^, which represented TUNEL positive nuclei of placenta in the late normal pregnancy and TUNEL negative placental nuclei in the first trimester normal pregnancy again leading us to say early apoptosis to be one of the cause of URSA^24^.

Abortions can occur due to innumerable causes but the abortions of unknown etiology are of grave concern. This study doesn’t claim the resulting pathway to be only one responsible, but its role can’t be denied in causation of URSA.

**Figure 7:**
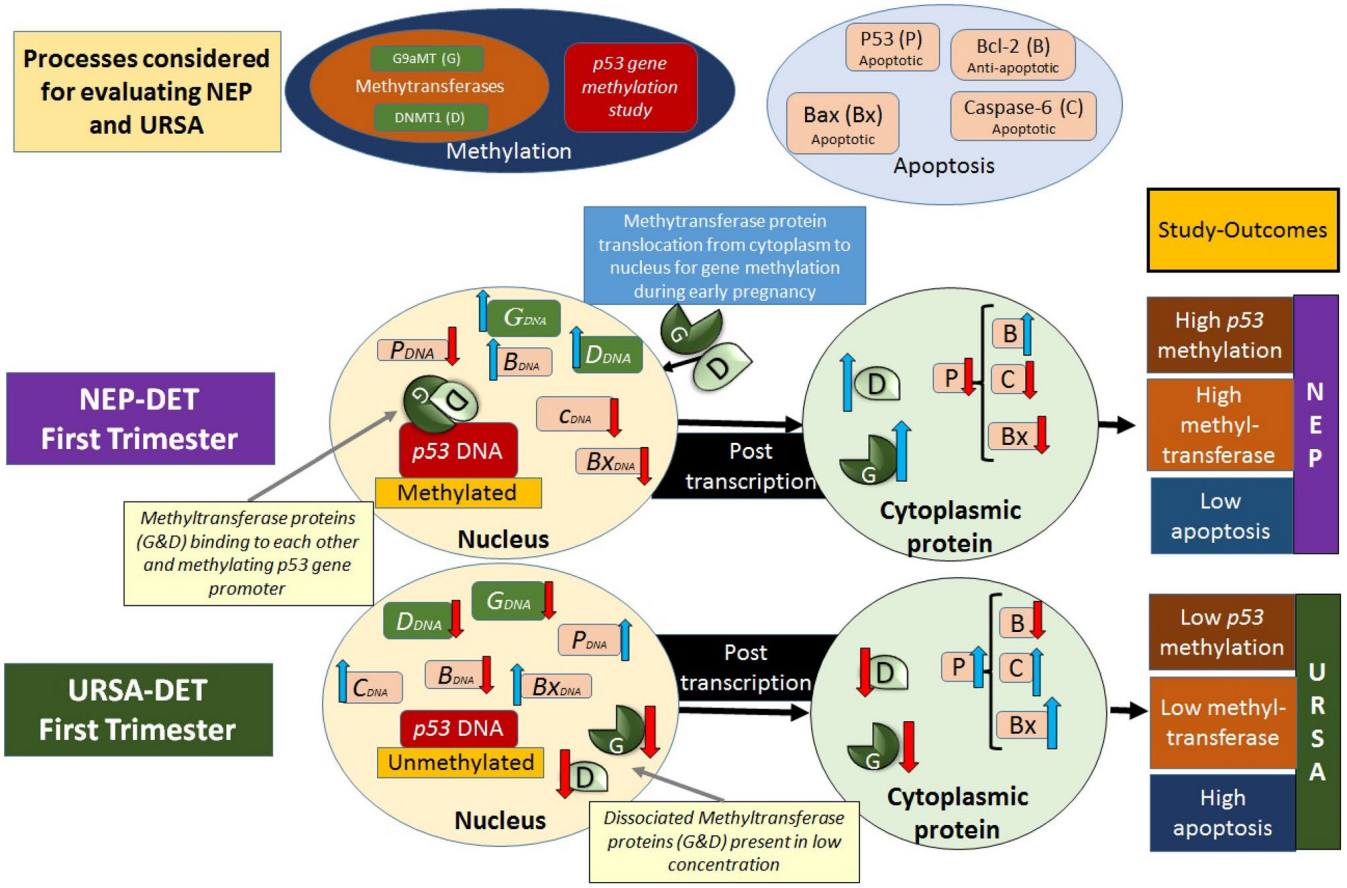
Diagrammatic representation of molecular expression during early pregnancy and unexplained abortions in human.

As per our knowledge, this study is the first to show causal relationship between anti-apoptotic (bcl-2), methyltransferases (G9a, and dnmt-1), methylation (p53), and apoptosis (TUNEL, Caspase-6, bax, and p53). Structural Equation Modelling estimated all the coefficient in the model to be the best fit in determining the causality through direct and indirect relationships (Tables 6 and 7, Fig. 6). The outcome of the study is shown in the illustrated diagrammatic representation of the molecular role during human early pregnancy and unexplained abortion in Fig. 7.

Interestingly, we may say that p53 methylation is always present whether high or low, but its level determines expression of p53 and further interplay of molecules. Lower level of p53 methylation in URSA leads to more apoptosis, while higher p53 methylation in NEP results to lower apoptosis. But apoptosis is an important phenomenon and shouldn’t be completely silenced. In conclusion, the most important achievement of the results of this study could be used in either designing/developing a diagnostic/prognostic kit or for developing a new drug. This drug may be any substance that could incur change in the methylation status of p53 level to treat the cases of URSA and/ or to develop anti-fertility drugs/abortifacients, to improve the quality of life in future to come without completely silencing it.

## Supplementary information

Supplementary file1

## References

[1] De Falco M (2001). Alteration of the Bcl-2: Bax ratio in the placenta as pregnancy proceeds. Histochem. J..

[2] Norimura T, Nomoto S, Katsuki M, Gondo Y, Kondo S (1996). p53-dependent apoptosis suppresses radiation-induced teratogenesis. Nat. Med..

[3] Sugino N (2000). Expression of Bcl-2 and Bax in the human corpus luteum during the menstrual cycle and in early pregnancy: regulation by human chorionic gonadotropin. J. Clin. Endocrinol. Metab..

[4] Uckan D (1997). Trophoblasts express Fas ligand: a proposed mechanism for immune privilege in placenta and maternal invasion. Mol. Hum. Reprod..

[5] Huppertz B, Frank HG, Kingdom JC, Reister F, Kaufmann P (1998). Villous cytotrophoblast regulation of the syncytial apoptotic cascade in the human placenta. Histochem. Cell Biol..

[6] Abrahams VM, Straszewski-Chavez SL, Guller S, Mor G (2004). First trimester trophoblast cells secrete Fas ligand which induces immune cell apoptosis. Mol. Hum. Reprod..

[7] Wouters MG (1993). Hyperhomocysteinemia: a risk factor in women with unexplained recurrent early pregnancy loss. Fertil. Steril..

[8] Practice Committee of the American Society for Reproductive, M. Evaluation and treatment of recurrent pregnancy loss: a committee opinion. *Fertil. Steril.* **98**, 1103–1111 (2012). 10.1016/j.fertnstert.2012.06.048 22835448

[9] Coulam CB, Kay C, Jeyendran RS (2006). Role of p53 codon 72 polymorphism in recurrent pregnancy loss. Reprod. Biomed. Online.

[10] Hirota Y (2010). Uterine-specific p53 deficiency confers premature uterine senescence and promotes preterm birth in mice. J. Clin. Invest..

[11] Choi HK (2003). Expression of angiogenesis- and apoptosis-related genes in chorionic villi derived from recurrent pregnancy loss patients. Mol. Reprod. Dev..

[12] Lea RG, AlSharekh N, Tulppala M, Critchley HOD (1997). The immunolocalization of bcl-2 at the maternal-fetal interface in healthy and failing pregnancies. Hum. Reprod..

[13] Fatima N (2011). Study of methyl transferase (G9aMT) and methylated histone (H3–K9) expressions in unexplained recurrent spontaneous abortion (URSA) and normal early pregnancy. Mol. Hum. Reprod..

[14] Esteve PO (2006). Direct interaction between DNMT1 and G9a coordinates DNA and histone methylation during replication. Genes Dev..

[15] Tachibana M (2002). G9a histone methyltransferase plays a dominant role in euchromatic histone H3 lysine 9 methylation and is essential for early embryogenesis. Genes Dev..

[16] Bill S (2000). Cause and Correlation in Biology. A User’s Guide to Path Analysis, Structural Equations, and Causal Inference.

[17] Rosa GJM (2011). Inferring causal phenotype networks using structural equation models. Genet. Sel. Evol..

[18] Kim J-Y, Namkung J-H, Lee S-M, Park T-S (2010). Application of structural equation models to genome-wide association analysis. Genom. Inform..

[19] Mi XJ, Eskridge KM, George V, Wang D (2011). Structural equation modeling of gene-environment interactions in coronary heart disease. Ann. Hum. Genet..

[20] Rao DC, Province MA (2000). The future of path analysis, segregation analysis, and combined models for genetic dissection of complex traits. Hum. Hered..

[21] Province MA (2003). Multivariate and multilocus variance components method, based on structural relationships to assess quantitative trait linkage via SEGPATH. Genet. Epidemiol..

[22] Li RH (2006). Structural model analysis of multiple quantitative traits. PLoS Genet..

[23] American College of Obstetricians and Gynecologists (ACOG) Practice Bulletin: No. 24, Feb 2001, Management of recurrent early pregnancy loss. *Int. J. Gynecol. Obste.* **78**, 179–190 (2002). 10.1016/s0020-7292(02)00197-2 12360906

[24] Fatima, N. *Study of Various Factors Involved in Recurrent Spontaneous Abortion in Females*. Aligarh Muslim University; https://hdl.handle.net/10603/56026 (2010).

[25] Kara F, Cinar O, Erdemli-Atabenli E, Tavil-Sabuncuoglu B, Can A (2007). Ultrastructural alterations in human decidua in miscarriages compared to normal pregnancy. Acta Obstet. Gynecol. Scand..

[26] Gompel A (1994). Bcl-2 expression in normal endometrium during the menstrual cycle. Am. J. Pathol..

[27] Koh EA, Illingworth PJ, Duncan WC, Critchley HO (1995). Immunolocalization of bcl-2 protein in human endometrium in the menstrual cycle and simulated early pregnancy. Hum. Reprod..

[28] Ci B (1967). Statistics in Biology.

[29] Laemmli UK (1970). Cleavage of structural proteins during the assembly of the head of bacteriophage T4. Nature.

[30] Berkova N (2001). Haptoglobin is present in human endometrium and shows elevated levels in the decidua during pregnancy. Mol. Hum. Reprod..

[31] Lowry OH, Rosebrough NJ, Farr AL, Randall RJ (1951). Protein measurement with the Folin phenol reagent. J. Biol. Chem..

[32] Baldi A (2000). Deafferentation-induced apoptosis of neurons in thalamic somatosensory nuclei of the newborn rat: critical period and rescue from cell death by peripherally applied neurotrophins. Eur. J. Neurosci..

[33] Watanabe H (1997). Bcl-2 and Fas expression in eutopic and ectopic human endometrium during the menstrual cycle in relation to endometrial cell apoptosis. Am. J. Obstet. Gynecol..

[34] Amatya VJ, Naumann U, Weller M, Ohgaki H (2005). TP53 promoter methylation in human gliomas. Acta Neuropathol..

[35] Zweig MH, Campbell G (1993). Receiver-operating characteristic (ROC) plots: a fundamental evaluation tool in clinical medicine. Clin. Chem..

[36] Jackson-Grusby L (2001). Loss of genomic methylation causes p53-dependent apoptosis and epigenetic deregulation. Nat. Genet..

[37] Haidacher S, Blaschitz A, Desoye G, Dohr G (1995). Immunohistochemical evidence of p53 protein in human placenta and choriocarcinoma cell lines. Hum. Reprod..

[38] Smith SC, Baker PN, Symonds EM (1997). Placental apoptosis in normal human pregnancy. Am. J. Obstet. Gynecol..

[39] Akcali KC, Khan SA, Moulton BC (1996). Effect of decidualization on the expression of bax and bcl-2 in the rat uterine endometrium. Endocrinology.

[40] Oltvai ZN, Milliman CL, Korsmeyer SJ (1993). Bcl-2 Heterodimerizes in-vivo with a conserved homolog, Bax that accelerates programmed cell-death. Cell.

[41] Piacentini M, Autuori F (1994). Immunohistochemical localization of tissue transglutaminase and Bcl-2 in rat uterine tissues during embryo implantation and post-partum involution. Differentiation.

[42] Wyllie AH (1994). Apoptosis. Death gets a brake. Nature.

