# Supplementary material for: Structural equation modelling analysis determining causal role among methyltransferases, methylation, and apoptosis during human pregnancy and abortion
Source: Sci Rep. 2020 Jul 24;10:12408. doi: 10.1038/s41598-020-68270-1 (PMC7381664; doi:10.1038/s41598-020-68270-1)
Supplement: Supplementary file 1 — Supplementary file1 [file 41598_2020_68270_MOESM1_ESM.docx]

**Structural Equation Modelling analysis determining causal role among *methyltransferases*, *methylation*, and *apoptosis* during human pregnancy and abortion**

**Nishat Fatima^1#,2^, S. H. Ahmed^4^, S. S. Chauhan^1^, M. Owais^2^ and S. M. F. Rehman^3#^**

^1^Department of Biochemistry, All India Institute of Medical Sciences (AIIMS), Ansari Nagar, New Delhi-110029, India

^2^Interdisciplinary Biotechnology Unit, Aligarh Muslim University (AMU), Aligarh-202001, Uttar Pradesh, India

^3^Department of Surgery, Dr. Ram Manohar Lohia Hospital and Post Graduate Institute of Medical Research Education and Research, New Delhi-110001, India

^4^Department of Management Studies, Indian Institute of Technology (IIT), New Delhi-110016, India

^#^ Equal first and corresponding authorship

^#^correspondence to [nishatbiotech@gmail.com](mailto:nishatbiotech@gmail.com)

**Supplementary Data**

**
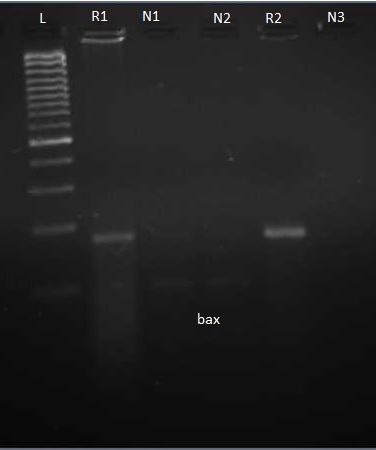
**

**
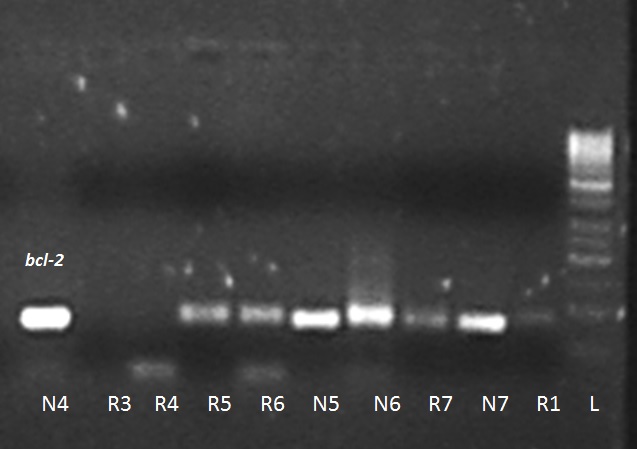
**

**
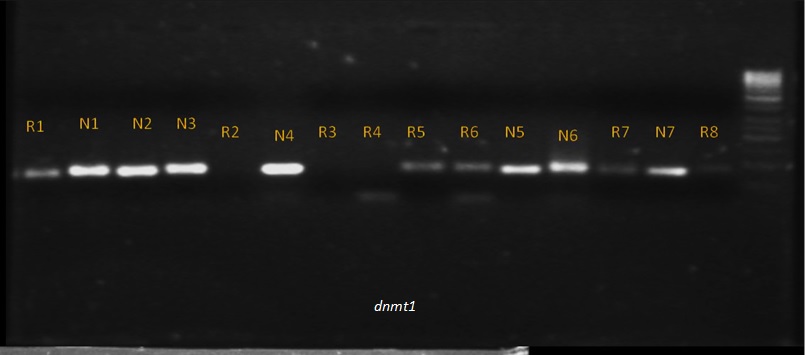
**

**
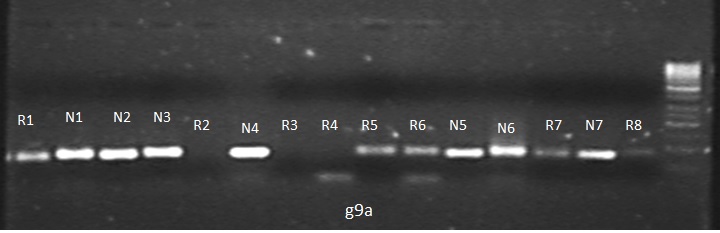
**

**
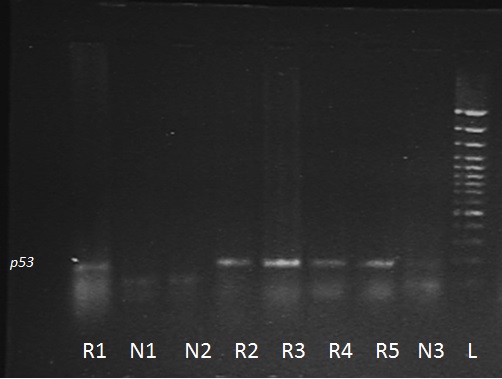
**
